# Using simulation to evaluate wildlife survey designs: polar bears and seals in the Chukchi Sea

**DOI:** 10.1098/rsos.150561

**Published:** 2016-01-27

**Authors:** Paul B. Conn, Erin E. Moreland, Eric V. Regehr, Erin L. Richmond, Michael F. Cameron, Peter L. Boveng

**Affiliations:** 1 Alaska Fisheries Science Center, National Marine Fisheries Service, NOAA, 7600 Sand Point Way NE, Seattle, WA 98115, USA; 2 US Fish and Wildlife Service, Marine Mammals Management, 1011 East Tudor Road, Anchorage, AK 99503, USA

**Keywords:** aerial survey, animal abundance, ice-associated seal, polar bear, species distribution model, survey design

## Abstract

Logistically demanding and expensive wildlife surveys should ideally yield defensible estimates. Here, we show how simulation can be used to evaluate alternative survey designs for estimating wildlife abundance. Specifically, we evaluate the potential of instrument-based aerial surveys (combining infrared imagery with high-resolution digital photography to detect and identify species) for estimating abundance of polar bears and seals in the Chukchi Sea. We investigate the consequences of different levels of survey effort, flight track allocation and model configuration on bias and precision of abundance estimators. For bearded seals (0.07 animals km^−2^) and ringed seals (1.29 animals km^−2^), we find that eight flights traversing ≈7840 km are sufficient to achieve target precision levels (coefficient of variation (CV)<20%) for a 2.94×10^5^ km^2^ study area. For polar bears (provisionally, 0.003 animals km^−2^), 12 flights traversing ≈11 760 km resulted in CVs ranging from 28 to 35%. Estimators were relatively unbiased with similar precision over different flight track allocation strategies and estimation models, although some combinations had superior performance. These findings suggest that instrument-based aerial surveys may provide a viable means for monitoring seal and polar bear populations on the surface of the sea ice over large Arctic regions. More broadly, our simulation-based approach to evaluating survey designs can serve as a template for biologists designing their own surveys.

## 1. Introduction

Population surveys are an important component of wildlife conservation and management, providing the data necessary to monitor population trends, inform management strategies, and measure impacts of management actions and other environmental perturbations. Such surveys can also be logistically demanding and expensive, particularly when study areas are large or when they are conducted in remote regions. Ensuring that such surveys yield defensible (i.e. unbiased) and useful (i.e. acceptable precision) estimates should be a focus of advance planning.

Wildlife surveys historically relied on design-based protocols for developing sampling frames (e.g. simple, stratified or systematic random sampling; [1]). If a survey can follow such a probabilistic design, estimators are guaranteed to be unbiased and there are relatively simple variance formulae for calculating anticipated precision. For instance, in distance sampling studies, pilot data informing expected encounter rates, detection probability and between-transect variance can be used to express anticipated precision as a function of survey effort [2].

Our experience with working in large, remote study systems suggests that deviations from pre-planned survey routes are often to be expected, as weather or logistical considerations often preclude surveying in predetermined locations. Employing design-based estimators in such cases may lead to bias and mis-stated precision. Alternatively, model-based approaches to estimation of wildlife parameters from survey data do not necessarily require strict adherence to a randomized sampling design. Model-based approaches are also gaining popularity because of the increased potential for assessing species–habitat relationships and producing maps of species distributions. For example, ecologists are increasingly fitting spatial models to distance sampling data [3–5], point count data [6,7] and replicated presence–absence data [8].

For model-based methods, analytical formulae relating survey effort to anticipated precision are usually unavailable. Further, bias may still result when the distribution of sampling effort is preferential (i.e. non-random and/or correlated with the distribution of the wildlife parameter of interest; [9,10]). When modelling quantities such as abundance that are non-negative, positive bias can also result when data are sparse or there is poor coverage, simply from the process of extrapolating modelled relationships (e.g. covariate effects or spatial random effects) to unsampled areas [11]. For these reasons, we argue that simulation studies are a critical first step in evaluating modern population surveys and that these should be conducted in advance, especially when a model-based approach to estimation is anticipated. Specifically, one can investigate how different levels of survey effort and effort allocation strategies affect bias and precision (e.g. [12–15]). Such exercises are especially important when surveys do not follow strict probability-based survey protocols owing to the potentially biasing effects of intentional or accidental preferential sampling [9,10].

In this study, we show how simulation can be used to evaluate alternative survey designs in planning surveys for polar bears (*Ursus maritimus*), bearded seals (*Erignathus barbatus*) and ringed seals (*Phoca hispida*) in the eastern Chukchi Sea (hereafter, CS). These species and study area exemplify the challenges of many wildlife surveys, as the study area is large, weather conditions are frequently poor, and there are only a limited number of air strips from which to conduct surveys. In addition, these species are of conservation concern. For instance, negative trends in seasonal Arctic sea-ice extent [16] have prompted concern for the viability of ice-associated marine mammals [17]. Worldwide, climatological projections suggest a 68% decline in optimal summer polar bear habitat by the end of the twenty-first century [18]. In two of the 19 recognized polar bear subpopulations [19], studies have linked declining sea-ice availability to declines in nutritional condition, reproduction, survival or abundance [20–24]. Two of the primary prey species of polar bears, bearded and ringed seals, also depend on sea ice for moulting, pupping and rest. Both polar bears and Arctic ringed seals are currently listened as threatened under the United States (US) Endangered Species Act [25,26]. The listing rationale for Arctic ringed seals was almost exclusively based on concern for future habitat declines, as current estimates indicate there are hundreds of thousands of ringed seals in the Bering and Chukchi seas alone [27,28].

Researchers have employed a variety of survey platforms to estimate the abundance and trends of Arctic marine mammals. Given their relatively low densities, abundance and trends of polar bears have primarily been estimated using labour intensive, multiyear mark–recapture studies [20,29–31]. Although distance-sampling aerial surveys are increasingly used to estimate polar bear abundance, such methods are most effective for subpopulations that spend the summer months on shore where they occur at higher densities and are relatively easy to sight [32,33]. By contrast, distance sampling surveys may be impractical over a substantial portion of the species’ range where polar bears spend the entire year on sea ice (but see [34]).

Aerial surveys of basking seals have been the primary tool for estimating phocid seal density over large spatial domains, often with corrections for imperfect detection and availability less than 1.0 [27,28,35,36]. Historically, aerial seal surveys were flown with on-board observers who counted and determined the species of detected seals. However, such surveys must be flown at relatively low speeds and altitudes, limiting the effective range of aircraft and potentially inducing escape behaviour that negatively biases abundance estimates [37]. Recent developments in instrument-based surveys (IBSs) that automate data collection (e.g. combining thermal video and high-resolution photography obtained from conventional fixed wing aircraft; [28,38]) appear to be a promising alternative for increasing survey efficiency and range, decreasing disturbance of animals, and reducing error relative to surveys with human observers. Given these improvements, an IBS may also have potential for monitoring polar bears, either complementing existing mark–recapture efforts or extending inference to populations that are less intensively studied.

In this study, we use simulation to assess the utility of an IBS for estimating the abundance of bearded seals, ringed seals and polar bears, using the eastern CS as an example study system. Populations of all three species in this area are subjects of conservation concern because of climatic warming [17], increased human activity [39], hydrocarbon exploration and development [40], and other factors.

This paper is organized as follows. First, we describe the proposed study area and technical specifications of the aerial survey platform. Next, we describe our simulation study in greater detail. Each simulation involves a number of steps, including: (i) using best available knowledge of species density and habitat preferences to simulate virtual populations, (ii) simulating application of alternative survey designs, and (iii) estimating animal abundance from simulated count data. After describing results of this study, we conclude with a discussion addressing the potential usefulness of our simulation approach. Specifically, we discuss the use of the IBS platform for surveying polar bears and seals in the CS and beyond. More broadly, we discuss the essential components of simulation studies when used as an omnibus procedure for evaluating efficacy of wildlife survey designs.

## 2. Material and methods

### 2.1 Study area

**Figure 1.**
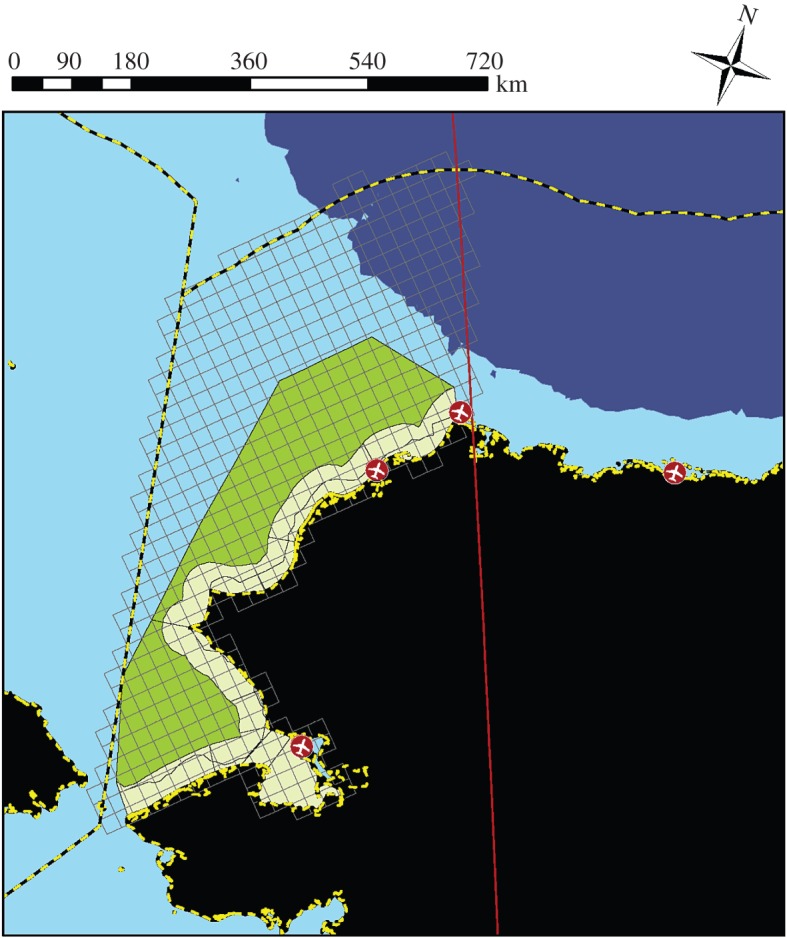
Potential study area for Arctic marine mammal surveys in the eastern Chukchi Sea off the coast of northwestern Alaska, USA. The study area is discretized into 625 km^2^ grid cells on a Polar Stereophonic projection (grey lines), commensurate with the resolution of prospective sea-ice covariate data. The boundaries of the study area are determined by the Bering Strait to the south, the US Exclusive Economic Zone to the west and north (yellow and black dashed line), and the 156° W longitude line to the west (red line). Land is indicated in black, with Alaska-based landing strips indicated as red circles. Water depths of more than 500 m are indicated in purple, while waters less than 500 m are light blue. Also shown are the 10 nearshore (yellow shading) and two offshore (green shading) strata used in previous seal surveys in this area [27].

We consider a potential application of an IBS in the eastern portion of the CS that occurs within US airspace (figure 1). This large study area (≈294 000 km^2^, or 21% larger than Great Britain) is equipped with two serviceable airports for prosecuting surveys (Barrow, AK, USA and Kotzebue, AK, USA), with several additional primitive airstrips available for emergency landings. Our objective will be to design surveys that permit reliable abundance estimation for focal species in this area during April and May. This timeframe is when ringed and bearded seals are engaged in ice-obligate behaviours such as pupping and moulting, and thus have the greatest probability of being hauled out on ice. Satellite tagging data suggest it is also before seals from the Bering Sea migrate northwards as sea ice retreats (J. London 2015, unpublished data).

### 2.2 Survey platform

**Figure 2.**
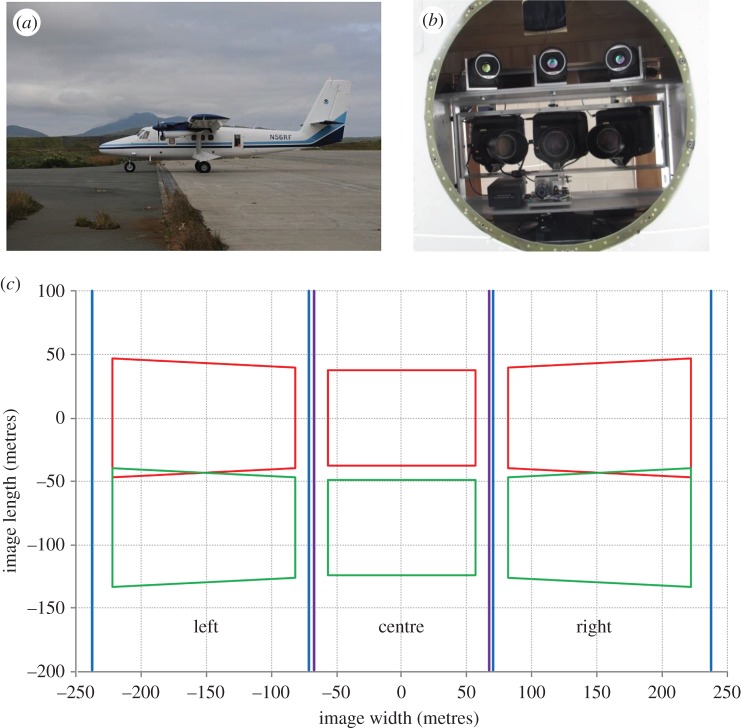
Equipment and camera specification for potential instrument-based surveys for polar bears and seals in the eastern Chukchi Sea. As with previous IBS surveys in the Bering Sea, a DeHavilland DHC-6 Twin Otter aircraft (*a*) would be equipped with three long wavelength infrared (LWIR) thermal cameras (FLIR SC645) and three high-resolution digital single-lens reflex (SLR) cameras (Canon 1Ds Mark III) mounted through its bellyport (*b*). Blue lines (*c*) indicate thermal camera swath width for each camera, while red and green polygons represent the expected footprint of SLR photographs. Flying at a target altitude of 300 m, this configuration results in a thermal swath width of 470 m of which automated digital photographs cover *ca* 84%.

We assumed that CS surveys would use similar technical specifications as a previous IBS of ice-associated seals in the Bering Sea [28]. In particular, we assumed that a DeHavilland DHC-6 Twin Otter aircraft would be equipped with three long wavelength infrared (LWIR) thermal cameras (FLIR SC645) and three high-resolution digital single-lens reflex (SLR) cameras (Canon 1Ds Mark III) mounted through its bellyport (figure 2). The thermal and SLR cameras would be equipped with 25 mm and 100 mm lenses, respectively, producing a thermal swath width of 470 m when flying at a target altitude of 300 m. Automated to take pictures every 1–1.4 s, digital photographs would cover 84% of the thermal swath (figure 2), and provide an estimated ground resolution of approximately 2 cm pixel^−1^. When the Twin Otter aircraft is equipped with an extra fuel tank, effective range given flight speed, altitude and safety considerations is roughly 900–1200 km, depending on the distance of a planned flight track to alternate (emergency) landing strips.

A previous double sampling experiment [28] indicated that seal detections with thermal cameras is quite high, with a detection rate of close to 94% (see ‘Estimating animal abundance’) for seals hauled out on ice. Non-detections were due mainly to human error, though seals recently emerged from water were also missed owing to reduced thermal radiation. Early investigations into infrared detection of polar bears on ice indicated that detecting wavelengths in the 8–14 μm band (LWIR) was a promising approach warranting further investigation [41]. Recent advances in LWIR detection technology and the software available to digitally interpret thermal data have led to improvements in polar bear detections. Anecdotal reports and recent research (e.g. [42]) confirm that even relatively affordable LWIR microbolometers have adequate sensitivity to detect polar bears on the ice despite the low emissivity [43] of polar bear hairs.

### 2.3 Data

**Figure 3.**
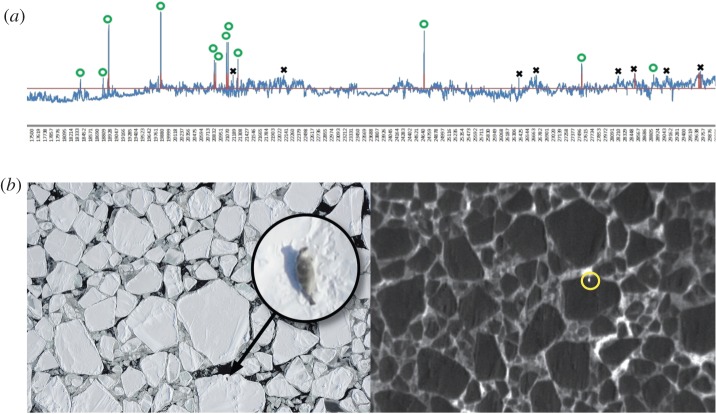
A depiction of the process with which animals are detected and counted using a coordination of thermal and SLR imagery. First, a time series of maximum pixel temperatures from thermal cameras are used to locate temperature peaks (*a*). Next, individual thermal video frames are reviewed to determine whether each peak is associated with a thermal signature of the size and shape that might be an animal (yellow circle in (*b*)). Such prospective ‘hot spots’ are denoted with a green ‘o’ in (*a*); pixel temperature peaks that did not meet this criterion are labelled with an ‘x.’ Finally, digital photographs with time stamps that match prospective hot spots are examined to confirm animal presence and determine species identity (*b*).

Rather than viewing terabytes of video and images manually, researchers review time series of maximum pixel temperatures and associated thermal video frames to identify ‘hot spots’, or heat signatures demonstrating a detectable difference in apparent temperature between an object and its background. Coordinated review of digital photographs with matching time stamps can provide information on species identity (figure 3).

### 2.4 Simulation study

We conducted a simulation study to examine the effectiveness of the IBS platform and associated hierarchical modelling framework for estimating abundance. Owing to long computing times for each individual model (see ‘Performance metrics and computing’), we limited this study to analysis of 100 hypothetical virtual populations. For each of these *n*=100 replicates, we (i) simulated the abundance and distribution of virtual populations of seals and polar bears, (ii) simulated datasets reflecting nine choices for survey effort and track allocation, and (iii) fit models with different combinations of explanatory variables for each dataset.

#### 2.4.1 Generating virtual populations

The best available data on seal abundance in the CS comes from aerial surveys flown in late May and early June in 1999 and 2000 [27], with density estimates available for 12 different spatial strata. Ringed seals had higher densities in nearshore fast and pack ice, with lower densities offshore, and were more common in the southern portions of the CS near Kotzebue Bay, AK, USA. Bearded seals had highest densities offshore in the southern portions of the study area [27], but had lower overall apparent density than ringed seals.

To generate virtual seal populations, we start by discretizing the study area into *J*=505 survey units, each of which is approximately 625 km^2^, the same resolution as commonly available sea-ice imagery (figure 1). We used strata-specific density estimates to define an initial density value for each cell, *d*_*j*_. In particular, we set *d*_*j*_ equal to seal density associated with the centroid of sample unit *j* (determined by locating the centroid of *j* on the density map generated by Bengtson *et al*. [27]). For survey units beyond the study area boundary used by Bengtson *et al*. [27], we applied density estimates from the nearest located strata.

Unlike seals, polar bear densities in the CS are largely unknown. Belikov [44] suggested that there were 2000–5000 polar bears in the CS region based on extrapolation of den surveys conducted on Wrangel Island. This estimate was later revised to 2000 by the Polar Bear Specialist Group of the International Union for the Conservation of Nature, based on expert opinion and concerns about the potential effects of habitat loss and human-caused mortality [45]. Given that the current IBS survey area covers roughly half of the CS polar bear subpopulation area, we considered *N*=1000 polar bears to be a reasonable approximation for simulation studies. We derived the relative probability of use *w*_*j*_ for each survey unit using scale-integrated resource selection functions for late April calculated from radiotelemetry data for polar bears over the period 2009–2012 [40]. We then determined the value of a fixed constant *c* such that $E(N)=\sum_{j}E(N_{j})=\sum_{j}cw_{j}=1000$, so that total expected (uncorrected) abundance (*E*(*N*)) was 1000 individuals. We then calculated *d*_*j*_ for polar bears as *d*_*j*_=*cw*_*j*_/625.

For seals, initial density values (*d*_*j*_) often included large differences in abundance between neighbouring survey units owing to the stark boundaries between survey strata in [27]. To make for a more continuous, biologically plausible expected density map, we smoothed initial density values by multiplying them by a (*J*×*J*) smoothing transition matrix, **W**. Elements of **W**, *w*_*ab*_, were determined as follows. First, diagonal elements *w*_*aa*_ were set to 2.0. Second, for all neighbours (i.e. when survey units *a* and *b* are adjacent to one another), *w*_*ab*_ was set to 1.0. All other entries of **W** were set to 0.0. Finally, elements of **W** were standardized to have each row of **W** sum to 1.0. A smoothed vector of densities ${\text{D}}^{*}=\{d_{1}^{*},d_{2}^{*},\ldots ,d_{K}^{*}\}$ was then computed as D*=**WD**, where **D**={*d*_1_,*d*_2_,…,*d*_*K*_}.

In order to generate meaningful differences in abundance among simulation replicates, we next introduced stochasticity in expected abundance (*λ*_*j*_). Our approach was to include moderate levels of extra-Poisson error and spatial autocorrelation to allow for simulated populations that were patchily distributed and overdispersed relative to the Poisson distribution, as is typical in many animal populations. Specifically, we calculated

$$\lambda_{j}=a_{j}\exp(\log(d_{j}^{*})+\eta_{j}+\epsilon_{j}),$$

where *a*_*j*_ gives the effective area of survey unit *j*, *η*_*j*_ denotes a mean zero, spatially autocorrelated random effect, and $\epsilon_{j}∼N(0,0.1)$ represents independent and identically distributed (iid) Gaussian error. We generated ***η***={*η*_1_,*η*_2_,…,*η*_*K*_} using an RSR-ICAR(*τ*_*η*_=5) distribution [28]. We set *a*_*j*_=625*R*_*j*_, where 625 (km^2^) was the area of each survey unit, and *R*_*j*_ gives the proportion of *j* that is composed of saltwater habitat. Over the course of *n*=100 simulation replicates, this approach yielded average expected population-level abundance of approximately 19 800 bearded seals, 380 000 ringed seals and 930 polar bears in surveyable portions of the study area.

#### 2.4.2 Simulating surveys

**Figure 4.**
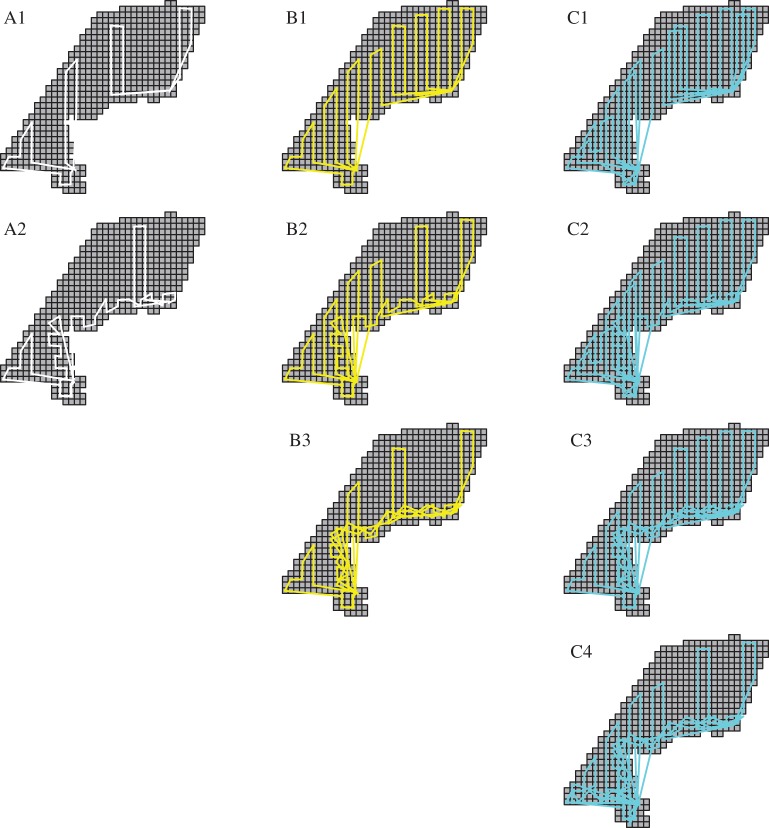
A collection of nine hypothetical aerial transect designs over the eastern Chukchi Sea, differing by: (i) number of flights (4, 8 or 12; displayed on columns), and (ii) the spatial distribution of tracks. The first row includes scenarios where only ‘long’ tracks are flown, trying to achieve more or less even coverage across the study area, while subsequent rows have an increasingly greater number of short tracks flown near the coast to target areas of higher seal densities.

We considered nine different possible aerial transect configurations that varied by total effort as well as effort allocation (figure 4). In particular, we included three different levels of effort: four flights, eight flights or 12 flights. Within each category, several different configurations were possible, allowing for relatively even coverage of the study area, or allowing for higher density of transects in areas of higher seal abundance. There is a theoretical basis for believing that the effort allocation will affect estimator performance. For instance, in the context of geostatistical models, relatively even coverage (spatial balance) tends to result in estimators with low bias and high precision [46,47]. However, in stratified random sampling [1] increases in estimator precision can be achieved by allocating more effort in high abundance strata. Stated another way, there may be little to be gained by allocating as much survey effort in places where focal taxa have low densities. By considering a range of effort allocation strategies, we hoped to examine whether a particular effort allocation strategy was advantageous over a range of estimation models (see ‘Estimating animal abundance’).

For each combination of virtual population (*n*=100) and survey allocation strategy (*n*=9), we simulated an IBS dataset. To begin, we calculated the proportion of area (*A*_*j*_) covered by digital photographs in each sampled survey unit *j*. Next, we generated the total number of animals of species *s* associated with the area surveyed in survey unit *j* as

$$n_{js}∼\mathrm{Poisson}(A_{j}\lambda_{js}).$$

Not all animals are detected in the surveyed area, however, owing to incomplete detection. As such, we generate the number of animals that are detected in digital photographs as

$$C_{js}∼\mathrm{Binomial}(n_{js},p_{js}),$$

where *p*_*js*_ reflects incomplete detection of species *s* in survey unit *j*. To generate data, we used a single value for *p*_*js*_=*a*_*js*_*p*_*s*_ for each species. Overall detectability, *p*_*js*_, is a product of both availability (the proportion of animals on ice while surveys are conducted) and detection probability of thermal cameras (the probability an animal is detected given that it is on ice and appears in digital photographs; *p*_*s*_). For bearded seals, we based availability, *a*_*js*_, on predicted haul-out probabilities estimated from satellite tagging records from bearded seals tagged in the CS (P. Conn 2014, unpublished data). Specifically, we set *a*_*js*_=0.48, which corresponds to predicted proportion hauled out on 8th May at 23.00 GMT (14.00 Alaska time). Surveys of seals in the CS would probably need to be prosecuted in early May, when a higher proportion of seals are available due to moulting and pupping, but before a large influx of seals into the study area from the Bering Sea occurs as seasonal ice melts. We do not currently have access to detailed telemetry records for ringed seals, and instead set *a*_*st*_=0.65 based on analyses reported by Bengtson *et al*. [27]. For polar bears, we set *a*_*st*_=1.0. Although some polar bears may be in the water at the time of surveys, this is not thought to be a large proportion and few data exist to further quantify this number. For polar bears and seals, we then applied a point estimate of *p*_*s*_=0.94, the point estimate of thermal detection probability from a double sampling study of seal photographs [28]. Note that this value implicitly includes different causes for non-detections (e.g. detection error, animals recently emerged from cold water, etc.). In absence of further information, we used this same value for polar bears.

#### 2.4.3 Estimating animal abundance

We used a hierarchical, Bayesian modelling framework to estimate animal abundance for each dataset. This was not strictly necessary, as other modelling approaches (e.g. generalized linear [48] or generalized additive [49] models) could have been used here. However, a Bayesian approach to estimation allows for straightforward modelling of spatial autocorrelation and provides a cohesive way to propagate uncertainty attributable to incomplete detection of animals.

The hierarchical modelling framework articulated here differs from that of Conn *et al.* [28], who modelled the latent species identity associated with each hot spot. This allowed them to model species for hot spots that did not have accompanying photographs and to account for species misclassification. However, this framework is extremely computationally intensive and is thus ill suited to large-scale simulation studies. In addition, several of these modelling features do not appear as useful for anticipated Chukchi IBS data, since (i) recent work on seal identification from digital SLR images indicates minimal misidentification between bearded and ringed seals [50], and (ii) limiting analysis to photographed animals will only require eliminating ≈20% of hot spot records. We thus adopted a simpler formulation for CS simulation analyses using aggregated count data that led to execution times roughly 100 times faster than those those reported in [28].

Following [28], variation in animal abundance is described using a spatial regression model. We start by writing a species-specific vector of expected abundances for all *J* survey units as

$$\lambda_{s}=\text{R}\;\exp({\text{X}}_{s}\beta_{s}+\eta_{s}+\epsilon_{s}),$$

where **R** is a vector giving the proportion of each survey unit that is composed of suitable habitat (i.e. that does not include land), **X**_*s*_ is a design matrix (e.g. [51]) that includes an intercept and desired covariates, ***β***_*s*_ is a vector of fixed effect regression parameters, ***η***_*s*_ is a vector of spatially autocorrelated random effects and ***ϵ***_*s*_ denotes a vector of independent, Gaussian errors. In the following simulation study, we use a reduced rank version of the intrinsic conditionally autoregressive prior [52] to impart spatial autocorrelation (see [28] for more information).

Of course, we do not observe true abundance for each survey unit. Survey units are not sampled in their entirety, nor do we necessarily observe all individuals in the sampled region. For this reason, the count of species *s* in survey unit *j*, *C*_*js*_, is conceptualized as arising according to a thinned Poisson process, where

$$C_{js}∼\mathrm{Poisson}(A_{j}p_{js}\lambda_{js}),$$

as in the previous section.

Unlike the data generating procedure where point estimates were used to generate count data, we wished to propagate uncertainty in detectability parameters when estimating abundance. One strategy for doing this is to specify informative prior distributions for detection parameters when conducting Bayesian inference. We employed the following procedure to generate prior distributions for *p*_*js*_. First, for all three species, we used data from a double sampling experiment [28] to produce a prior distribution for *p*_*s*_. This experiment yielded 70 seal detections using independent searches of photographs. Of these, 66 were detected using thermal cameras from the same target altitude as planned for CS surveys. We used these data to specify a Beta prior distribution for *p*_*s*_ for all three species:

$$p_{s}∼\mathrm{Beta}(71,5).$$

Next, for bearded seals, we generated a prior distribution for *a*_*js*_ from previously analysed satellite telemetry records, using the same procedure as described in [28]. For purposes of this analysis, we used the predicted proportion of bearded seals hauled out on 8th May at 23.00 GMT (14.00 Alaska time) (the same date and time used for point estimates when generating IBS data). For ringed seals, this distribution was rescaled to have a mean of 0.65, effectively assuming similar uncertainty about predicted proportions hauled out between the two species. For polar bears, we simply set *a*_*js*_ to 1.0, thus assuming that polar bears spend a negligible portion of time in the water and underwater, and are effectively always available to be detected. Finally, we generated a simple Monte Carlo sample of 1000 from the prior distribution of *p*_*js*_, [*p*_*js*_], by independently sampling from the independent distributions [*a*_*js*_] and [*p*_*s*_] and multiplying each replicates values together. These samples were used as the prior distribution for *p*_*js*_.

Conducting a Bayesian analysis requires a method for summarizing the posterior distribution of model parameters, where the posterior distribution is proportional to the prior distribution times the likelihood. This is difficult to do directly, so practitioners have developed simulation-based approaches such as Markov chain Monte Carlo (MCMC) to sample the posterior. Here we used a variant of MCMC, namely Metropolis-within-Gibbs sampling [53] to summarize the posterior by cyclically drawing from each parameter’s so-called full conditional distribution. In particular, we used the same Metropolis-within-Gibbs sampling procedure as specified by Conn *et al*. [28] (omitting updates for true species, species misclassification parameters, and individual covariate distributions). This procedure required us to specify prior distributions for several additional sets of parameters. We chose the same set of vague priors as implemented by Conn *et al*. [28], namely:

$$\begin{array}{rl} \left[\beta_{s}\right] & =MVN(\text{0},{\left(0.01{\text{X}}_{s}^{\prime }{\text{X}}_{s}\right)}^{-1}),\\ \left[\tau_{\eta }\right] & =\mathrm{Gamma}(1.0,0.01)\;\mathrm{and}\\ \left[\tau_{\epsilon }\right] & =\mathrm{Gamma}(1.0,0.01). \end{array}$$

The prior for regression parameters, [***β***_*s*_], is a diffuse multivariate normal distribution scaled so that the effect of the prior is similar regardless of the absolute value of the covariates used in the analysis. The parameters *τ*_*η*_ and *τ*_*ϵ*_ represent precision for spatial random effects (when modelled), and extra-Poisson error, respectively. Small values of precision (e.g. 0.1) result in high levels of spatial autocorrelation or overdispersion (for *τ*_*η*_ and *τ*_*ϵ*_, respectively), while large values (e.g. 100) represent low spatial autocorrelation or overdispersion. Our gamma prior (i) puts substantial mass on all reasonable parameter values, and (ii) has roughly uniform mass near the origin, which helps prevent pathological behaviour exhibited by other commonly used gamma priors (e.g. [54]).

To emulate realistic analyses performed on IBS data, we compiled a number of geographical covariates that might be useful predictors of animal abundance, including: (i) distance from each survey unit to the mainland of Alaska, USA (‘dist.land’), (ii) vertical distance along the survey grid (‘northing’), and (iii) horizontal distance along the survey grid (‘easting’). These covariates were intended to capture large-scale spatial trends in abundance via a response surface; note that in future analyses with real data it would probably be beneficial to consider additional ecologically relevant covariates (e.g. sea ice concentration, distance from ice edge, bathymetry). All geographical covariates were computed relative to the centroid of each 25×25 km survey unit on a Polar Stereographic projection. When modelled, we included linear effects of all geographical covariates, as well as a square root transform of ‘dist.land’ and an easting × northing interaction. In several estimation models, we also used the 12 strata employed by Bengtson *et al*. [27] as a post hoc categorical variable (‘strata’; figure 1). Finally, we computed a covariate that summarized the relative density of survey effort across the survey area (‘samp.dens’). Recent investigations have indicated potential for bias in model-based analyses when survey effort is not randomized [9], but controlling for the relative density of survey effort has been shown to mitigate bias in some applications [10]. To compute the effort covariate, we first summarized total survey effort (i.e. area sampled) by survey unit. Next, we used the function ‘KernSur’ in the GenKern R library (with default bandwidths), to compute a two-dimensional kernel density estimate (KDE) of observed effort at each of the survey unit centroids. We then standardized each KDE by dividing by its mean to produce the ‘samp.dens’ covariate.

We examined trace plots and standard MCMC diagnostics (e.g. Gelman-Rubin diagnostics; [53]) for several representative model runs to help guide the length of MCMC chains used in the simulation study. In particular, these diagnostics helped us to ensure that MCMC chains had reached their stationary distribution and provided guidance on sample sizes needed to ensure reasonable inference. Each MCMC run began with 3000 iterations used to help tune MCMC updates to achieve target acceptance rates between 30 and 40%, as suggested by Gelman *et al*. [53]. For both seal species, an additional 60 000 MCMC iterations were performed, with the first 10 000 discarded as a burn in. Saving every 25th iteration to reduce disc storage resulted in 2000 samples from the joint posterior distribution for each model. Owing to considerably sparser data, polar bear models were run for much longer. Following the initial 3000 iteration adapt phase, polar bear models were run for 450 000 iterations, with the first 50 000 iterations discarded as a burn in. We saved data from every 200 iterations to once again arrive at 2000 posterior samples.

**Figure 5.**
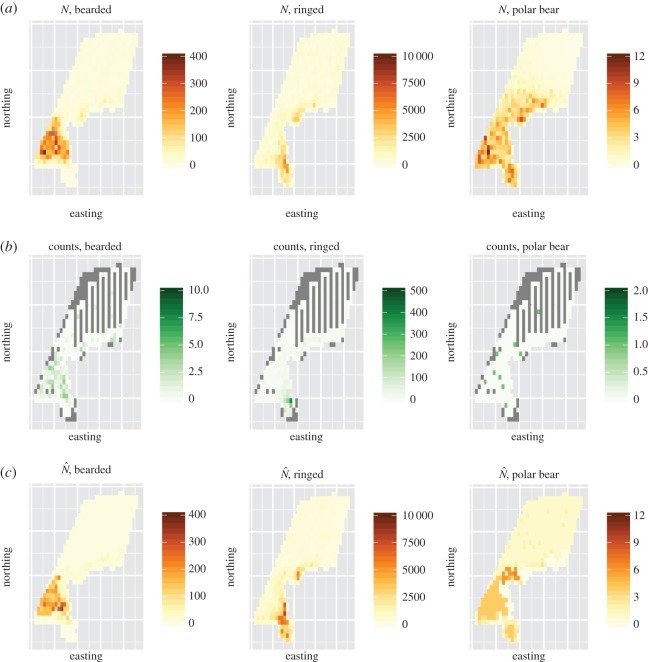
Visualization of a single simulation replicate of instrumental-based surveys for seals and polar bears in the Chukchi Sea. First, a realization of abundance (*N*) is simulated for each species (*a*). Next, virtual transect surveys are conducted, yielding counts for each species (*b*). Note that grey cells are unsurveyed. Finally, abundance is estimated ($\hat{N}$; *c*), and compared to simulated abundance to tabulate performance metrics.

We initially attempted to fit a total of 12 models to each seal count dataset and eight models to each polar bear dataset. However, issues with numerical overflows and lack of parameter identifiability prevented us from fitting all models to all datasets (table 1). For instance, owing to the small number of encounters, polar bear models were often unstable when only four flights were conducted or when spatial random effects were employed. We did not perform any simulations at these design points. An example of a single simulation replicate is provided in figure 5.

**Table 1.** Proportion relative bias for the total abundance estimator ($\hat{N}$), as estimated from different models (indicated on rows), and as a function of different transect configurations (columns). (Each value represents median proportional bias of *n*= 100 simulation replicates, where the posterior predictive mean of $\hat{N}$ is used as a point estimator. Models could include landscape-level covariates (collectively referred to as ‘covs’), geographical stratum (‘stratum’), a measure of sampling intensity (intended as a potential fix for preferential sampling; ‘samp.dens’) and spatially autocorrelated ‘RE’. Different flight configurations are displayed in figure 4; the first arabic symbol in the flight name relates to the number of flights (4, 8 or 12 flights), while the second reflects the amount of effort devoted to nearshore surveys (1=no coastal surveys, 2=2 coastal surveys, 3=4 coastal surveys, 4=6 coastal surveys). Blank entries indicate cases where estimates were numerically unstable or models were otherwise overparametrized.)

	4 flights		8 flights			12 flights			
model	A1	A2	B1	B2	B3	C1	C2	C3	C4
*(a) bearded seals*									
covs	−0.03	0.54	−0.04	0.03	0.08	−0.00	0.06	0.07	−0.01
covs + samp.dens	0.18	0.15	−0.05	−0.08	−0.07	−0.06	−0.04	−0.02	−0.10
stratum		−0.07	−0.05	−0.05	−0.05	−0.05	−0.04	−0.05	−0.06
covs + stratum		0.14	−0.04	−0.07	−0.04	−0.05	−0.04	−0.03	−0.07
stratum + samp.dens		−0.07	−0.04	−0.07	−0.04	−0.06	−0.06	−0.01	−0.09
covs + stratum + samp.dens		0.12	−0.03	−0.04	0.01	−0.03	−0.05	−0.01	−0.07
covs + RE	−0.03	0.59	−0.03	0.01	0.04	−0.03	−0.01	0.01	−0.02
stratum + RE		−0.07	−0.06	−0.07	−0.05	−0.09	−0.05	−0.05	−0.07
stratum + samp.dens + RE		−0.08	−0.05	−0.06	−0.03	−0.06	−0.05	−0.02	−0.10
*(b) ringed seals*									
covs	−0.01	0.19	0.04	0.09	0.13	0.04	0.06	0.10	0.07
covs + samp.dens	0.14	0.21	0.04	−0.00	−0.01	0.02	0.02	0.00	−0.02
stratum		0.09	−0.00	0.03	0.05	−0.02	−0.01	0.03	0.02
covs + stratum		0.10	−0.01	0.01	0.03	−0.00	0.00	0.02	0.00
stratum + samp.dens		0.05	−0.00	−0.01	−0.03	−0.02	−0.02	−0.01	−0.03
covs + stratum + samp.dens		0.10	−0.00	−0.02	−0.01	−0.01	−0.01	−0.01	−0.04
covs + RE	−0.02	0.13	−0.00	0.01	0.04	−0.01	−0.00	0.01	−0.01
stratum + RE		0.09	−0.00	0.03	0.06	−0.02	−0.00	0.02	0.02
stratum + samp.dens + RE		0.05	0.00	−0.02	−0.03	−0.02	−0.02	−0.01	−0.04
*(c) polar bears*									
covs			0.01	−0.11	0.07	−0.04	−0.09	−0.05	−0.07
covs + samp.dens						0.02	0.02	−0.08	−0.04
stratum			−0.11	−0.07	0.19	−0.08	−0.07	−0.05	0.11
stratum + samp.dens			0.38	−0.05	0.04	−0.04	−0.01	−0.04	0.07

#### 2.4.4 Performance metrics and computing

For each model fit to count data, we computed proportional bias, precision (coefficient of variation; CV), root mean square error (RMSE) and 90% credible interval coverage (CIcov). Bias, CV and RMSE were calculated relative to the posterior predictive mean for total abundance. We report median values (over all *n*=100 simulation replicates) for bias and CV to lessen the impact of outliers. For instance, several simulations for polar bears with low effort and higher levels of model complexity resulted in clearly unrealistic estimates in the tens of thousands. Basing model performance statistics on the median reduced the influence of outliers that represented particularly poor estimation. To calculate CIcov, we determined the proportion of simulations for which the true value for total abundance (*N*) was between the 5th and 95th posterior quantiles of estimated abundance.

We performed all analyses in the R programming environment [55], and developed an R package, ChukchiPower, to house all simulation and analysis code. This package has been published online [56] and is also available on github at https://github.com/pconn/ChukchiPower.

Requisite computing time for each MCMC run varied depending on the amount of data, model input configuration and species (with polar bears requiring a greater number of MCMC iterations). With fewer data (e.g. four flights) and a simpler model structure (e.g. no spatial random effects), a single MCMC run for a seal species could be accomplished in less than a minute on a Dell Precision laptop with 3.0 GHz processors. With 12 flights and models with spatial random effects, individual simulations took up to 14 min. In total, 17 800 analyses of simulated datasets were conducted, requiring approximately 100 CPU-days of computing time.

## 3. Results and discussion

We evaluated the impact of alternative study designs on bias and precision of model-based abundance estimators. Simulations revealed that four flights are too few to produce viable estimates of abundance for the three species studied, as median absolute proportional bias for seals was frequently >0.1 and as high as 0.59, depending on the estimation model (table 1). CV was also greater than our target of 0.2 for bearded seals (table 2).

**Table 2.** CV for the total abundance estimator ($\hat{\mathrm{SE}}(\hat{N})/\hat{N}$), as estimated from different models (indicated on rows), and as a function of different transect configurations (columns). (Each value represents the median CV over *n*=100 simulation replicates, where the posterior predictive mean of $\hat{N}$ is used as a point estimator. Models could include landscape-level covariates (collectively referred to as ‘covs’), geographical stratum (‘stratum’), a measure of sampling intensity (intended as a potential fix for preferential sampling; ‘samp.dens’) and spatially autocorrelated ‘RE’. Different flight configurations are displayed in figure 4. Blank entries indicate cases where estimates were numerically unstable or models were otherwise overparametrized.)

	4 flights		8 flights			12 flights			
model	A1	A2	B1	B2	B3	C1	C2	C3	C4
*(a) bearded seals*									
covs	0.21	0.28	0.16	0.16	0.17	0.13	0.14	0.15	0.15
covs + samp.dens	0.33	0.27	0.16	0.16	0.18	0.13	0.13	0.15	0.15
stratum		0.19	0.15	0.15	0.16	0.13	0.13	0.14	0.14
covs + stratum		0.34	0.15	0.15	0.16	0.13	0.13	0.14	0.14
stratum + samp.dens		0.19	0.15	0.15	0.17	0.13	0.13	0.14	0.14
covs + stratum + samp.dens		0.39	0.16	0.16	0.18	0.13	0.13	0.15	0.15
covs + RE	0.21	0.31	0.15	0.16	0.18	0.13	0.13	0.14	0.15
stratum + RE		0.19	0.15	0.15	0.16	0.13	0.13	0.14	0.14
stratum + samp.dens + RE		0.20	0.16	0.16	0.18	0.13	0.13	0.14	0.14
*(b) ringed seals*									
covs	0.11	0.12	0.10	0.10	0.10	0.10	0.10	0.10	0.09
covs + samp.dens	0.14	0.13	0.11	0.10	0.10	0.10	0.09	0.10	0.09
stratum		0.11	0.10	0.09	0.10	0.09	0.09	0.09	0.09
covs + stratum		0.12	0.10	0.10	0.10	0.09	0.09	0.09	0.09
stratum + samp.dens		0.10	0.10	0.10	0.10	0.09	0.09	0.09	0.09
covs + stratum + samp.dens		0.12	0.10	0.10	0.10	0.10	0.09	0.09	0.09
covs + RE	0.11	0.12	0.10	0.10	0.10	0.09	0.09	0.09	0.09
stratum + RE		0.11	0.10	0.10	0.10	0.09	0.09	0.09	0.09
stratum + samp.dens + RE		0.11	0.10	0.10	0.10	0.09	0.09	0.09	0.09
*(c) polar bears*									
covs			0.40	0.41	0.39	0.31	0.30	0.32	0.30
covs + samp.dens						0.35	0.35	0.35	0.32
stratum			0.38	0.37	0.35	0.30	0.28	0.30	0.30
stratum + samp.dens			0.97	0.42	0.42	0.35	0.34	0.35	0.32

Eight flights produced CV values for both seal species that were all less than 20%, although CV for polar bears was much higher (35%<CV<106%; table 2) and with biases that were of concerning magnitude (e.g. up to 0.38; table 1). Going to 12 flights improved precision even further with <15% for both seal species for all flight combinations and estimation models considered, and reduced CV for polar bears to a level with more applied value (e.g. 28%<CV<35%; table 2).

Limiting simulations to those with eight or more flights, proportional bias was often slightly negative for bearded seals, roughly zero for ringed seals, and mixed for polar bears (table 1). For the 12 flight scenarios, biases were typically less in scenarios C1–C3 than for C4, indicating that C4 may be a poor effort allocation strategy. Evidently, moderately elevating levels of effort in nearshore strata does not greatly help or hurt abundance estimation, but too much effort in nearshore strata can hurt the quality of inference (C4 has the highest levels of effort allocation to nearshore strata; figure 4).

The model used for estimation did have some impact on estimates. In general, simple models including only landscape covariates tended to produce estimates with higher bias than more complex models. For bearded seals, including the sampling density as a predictive covariate (via ‘samp.dens’) did not appear to be a useful strategy for reducing bias associated with preferential sampling. For polar bears, models with sampling density often performed better than those without; absolute bias was reduced considerably for seven out of eight flight combinations (table 1). Including such a variable is important if flight tracks are placed so that they either over- or under-sample polar bears relative to unsurveyed cells with similar predictive covariates.

RMSE is viewed by some as the most useful descriptor of estimator performance as it combines notions of bias and precision. Of eight-flight scenarios B1–B3, B1 had better overall RMSE scores for seals than the other options (electronic supplementary material, table S1). For the 12-flight scenarios, performance was not as clear cut, with C1–C3 clearly having lower (better) RMSE scores than C4, but the superiority of RMSE scores among C1–C3 depended on species and estimation model.

Credible interval coverage was greater than nominal for bearded and ringed seals, and close to nominal for polar bears (electronic supplementary material, table S2). For seals, this was probably an artefact of us using a point estimate for availability when simulating data, but assuming increased uncertainty about its value during estimation. Thus, we think estimated precision levels are probably accurate (i.e. CIcov greater than nominal is not necessarily indicative of estimated variances that are too high).

To compare CV from our study with other published field efforts, we used the approximate relationship $\mathrm{CV}\approx (\mathrm{High}-\mathrm{Low})/(4\hat{N})$ to calculate CV for studies where only confidence or credible intervals were reported. Here, ‘High’ and ‘Low’ give higher and lower 95% confidence/credible interval limits, respectively. For ringed seals, previous fixed-wing aerial distance sampling surveys in the CS [27] reported CVs of 0.19 and 0.12, which is similar or worse precision than we projected in our simulation study. In an IBS aerial survey in the eastern Bering Sea, Conn *et al.* [28] achieved a CV of close to 0.10 for bearded seals using 10 flights of similar range to those considered here. This is slightly more precise than projected for bearded seals in the CS. By contrast, Ver Hoef *et al.* [36] obtained a model-based CV for bearded seals of 0.55 in the Bering Sea using distance-sampling helicopter surveys, which is substantially worse precision than obtained here. This difference probably results from a number of factors, including: (i) lower coverage of helicopter surveys, (ii) additional variance from estimating the detection function in distance sampling surveys, and (iii) an attempt being made in [36] to account for changing sea ice conditions.

Previous surveys for polar bears have yielded much better CV values than the CVs projected in this paper. For instance, Bromaghin *et al.* [29] obtained an abundance estimate for the Southern Beaufort Sea subpopulation in 2010 with a CV of 0.17, but this required data from a 10-year, intensive mark–recapture study. Also, estimates of abundance from open-population mark–recapture studies apply to the ‘superpopulation’ (i.e. the group of animals with a non-negligible probability of moving through the sampling area), which can be difficult to define and may be different from the population of interest from a biological or management perspective [24]. By contrast, aerial surveys permit estimation of the population of animals located within the study area while the survey is being conducted.

Using helicopter-based distance sampling surveys, Aars *et al.* [34] obtained a CV of 0.16 for the Barents Sea polar bear subpopulation. However, they flew almost double the length of transects than considered here (20 975 km compared to 11 760 km in our 12 flight scenario) and surveyed a much denser population. For instance, Aars *et al.* [34] encountered a total of 263 bears, while we estimated that we would probably encounter just 20–30 bears in our surveys. Similarly, Stapleton *et al.* [32] obtained a CV of 0.16 for the Western Hudson Bay subpopulation; however, this survey was conducted in the ice-free summer period when the population had moved exclusively onto land and were therefore more congregated and visible. Applying similar survey methods in the CS could be expected to result in substantially lower precision. In addition, the reduction in speed, altitude and range required for using distance sampling from helicopters would probably make this sampling approach infeasible for the CS subpopulation. In a simulation study specifically evaluating distance sampling from two helicopters based on an ice breaker to estimate polar bear abundance in the CS, Nielsen *et al.* [13] found that for a population of *N*=2000, distance sampling abundance estimators had moderate (≈10%) positive bias and a CV of 25–40%. On the surface, this estimate looks promising. However, in addition to a high projected cost (1.6–3.0 million USD), this study used the same set of resource selection function (RSF) estimates in two ways: once to generate polar bear locations, and a second time to extrapolate estimates to unsurveyed locations. Such a double use of data may have led to overly optimistic estimates, as it suggests analysts have precise knowledge of the process by which bears distribute themselves along the sea ice. By contrast, we made no such assumption in this study, as the RSF estimates we used to help generate bear locations were not included as explanatory covariates within estimation models.

## 4. Conclusion

For seals and polar bears, IBS surveys appear a promising avenue for monitoring or supplementing other research on population abundance. For bearded and ringed seals, eight IBS flights over the eastern CS appear sufficient for estimating abundance with a low degree of bias and high degree of precision (CV<20% for both species). This is encouraging, as precise estimates of seal abundance and distribution will be important for gauging the ultimate effects of climate change and other human influences on seal populations and for evaluating recent threatened species protections (e.g. [26]).

For polar bears, 12 flights would probably lead to a CV between 28 and 35%, which would provide more information on population size than is currently available and could help complement multiyear surveys that provide more detailed information on population ecology and demography (e.g. mark–recapture or telemetry surveys) but do not provide useful estimates of abundance. Estimates of absolute abundance are directly relevant to some management questions (e.g. estimation of sustainable harvest levels), and can help anchor integrated population models for bear populations that combine data from disparate sources [57], improving the quality of inference. Despite the relatively low precision compared with other published aerial survey estimates, we are encouraged by our results, especially because (i) polar bear densities for the eastern CS are much lower than other locations where distance sampling surveys have been conducted, and (ii) the range, speed and altitude of the IBS surveys proposed in this paper allow for more extensive coverage of the survey area than do helicopter-based distance sampling surveys (e.g. [34,32]).

Our approach in this paper was to use relatively simple models for animal abundance which included the capacity for overdispersion (relative to the Poisson) but did not include possible zero inflation. This may be an important consideration for more abundant species (e.g. ringed seals), and would serve to decrease precision relative to what has been presented in this paper. Owing to differences in performance among estimation models, it may also be important to include model uncertainty through model averaging, which also would probably decrease precision on resultant estimators. Similarly, our simulation design allowed for a moderate level of patchiness in abundance through spatial random effects but did not consider higher levels of patchiness. Thus, estimates of precision obtained in this paper should be interpreted conservatively.

The study area evaluated in this paper consisted of the eastern (US) portion of the CS, which is only a portion of the Chukchi region relevant to management and conservation of Arctic marine mammals. Although not described here, a similar IBS survey of the western (Russian) portion of the CS is also being planned, with both surveys (east and west) set to commence in April 2016. Applying similar levels of survey effort and track distribution to the western CS should allow for viable abundance estimates and maps for the CS as a whole. More broadly, we are optimistic that IBS surveys will be useful for quantifying the abundance of Arctic marine mammals in sea-ice environments. These surveys may complement existing efforts in intensively studied populations or may allow investigators to extend the spatial scope of inferences being made to populations that are more difficult to survey owing to logistical limitations. To obtain robust estimates from such surveys, future effort should be devoted to estimating components of detection for polar bears, such as availability and detection probabilities from thermal cameras. The latter could be accomplished through experimental trials where the aerial survey platform is flown over polar bears on ice that are detected visually. Measuring separate detection rates for polar bears is especially important given that polar bear hair masks their heat signature more than other species [43]. Reductions in detection rates and increases in uncertainty associated with detection from the nominal values assumed in this paper would decrease precision of abundance estimates.

We suggest that analyses presented here serve as a template for evaluating the potential for IBS surveys to estimate abundance of other Arctic marine mammal populations. More broadly, our general three-step approach of simulating virtual populations, simulating surveys and estimating animal population parameters can be used to help compare alternative survey designs in other settings. To employ this three-step approach, several basics are required. First, investigators need to have a reasonable map of species density. It need not be exact; expert opinion can often be used in this step if no other data are available, or several such maps can be created that embody alternative states of nature. Second, alternative survey specifications (or computer code for generating surveys) must be available and georectified. Finally, landscape-level covariates are useful for formulating estimation models that embody different levels of complexity. With these three elements in hand, construction of a simulation analysis is conceptually straightforward, although some expertise with geospatial analysis and computer coding will usually be needed (e.g. to intersect surveys with virtual animal populations and to loop over simulation replicates).

Several elements of our study should routinely be included in similar simulation analyses. First, inclusion of spatial random effects when simulating virtual populations can emulate the patchiness that is typical of animal populations; ignoring this effect will tend to lead to estimates of survey efficiency that are too optimistic. Second, considering a suite of estimation models can help investigators decide on a realistic level of model complexity. Models that are too simplistic will do a poor job at describing variation in animal abundance across a landscape; models that are too complex will have lower precision and may suffer from inaccurate and biased extrapolations of abundance to unsampled areas [11]. Third, we suggest using different mechanisms for generating virtual populations and estimating animal abundance. Including the same covariates or functional forms in the data-generating and estimation models will probably paint an overly optimistic picture of estimator performance. Finally, we encourage analysts to incorporate sound design-based principals into their study design whenever possible. Although it may be possible to detect and correct for preferential sampling as part of the modelling process (e.g. using a covariate reflecting density of sampling), such models can sometimes be unstable and design-based principles such as randomization can help avoid preferential sampling in the first place. More sophisticated approaches for modelling preferential sampling effects is a focus of current research.

## Supplementary Material

Supplementary simulation study results. Tables describing root mean square error and 90% credible interval coverage for seal and polar bear abundance, as estimated from simulations of instrument-based aerial surveys in the eastern Chukchi Sea.

## Data accessibility

We developed an R package, ‘ChukchiPower,’ to house all data and code needed to recreate our analyses. This package has been published to fig**share** [56].
