# Supplementary material for: Using simulation to evaluate wildlife survey designs: polar bears and seals in the Chukchi Sea
Source: R Soc Open Sci. 2016 Jan 27;3(1):150561. doi: 10.1098/rsos.150561 (PMC4736938; doi:10.1098/rsos.150561)
Supplement: Supplementary simulation study results. Tables describing root mean square error and 90% credible interval coverage for seal and polar bear abundance, as estimated from simulations of instrument-based aerial surveys in the eastern Chukchi Sea. [file rsos150561supp1.pdf]

Evaluating instrumental aerial surveys as a platform for estimating polar bear and seal abundance in the eastern Chukchi sea: Supplement

Paul B. Conn, Erin E. Moreland, Eric V. Regehr, Erin L. Richmond, Michael F. Cameron, and Peter L. Boveng

Tables

Table S1. Root mean squared error (RMSE) for the total abundance estimator ($\hat{N}$), as estimated from different models (indicated on rows), and as a function of different transect configurations (rows). Each value represents RMSE over $n = 100$ simulation replicates, where the posterior predictive mean of $\hat{N}$ is used as a point estimator. Models could include landscape level covariates (collectively referred to as "covs"), geographical stratum ("stratum"), a measure of sampling intensity (intended as a potential fix for preferential sampling; "samp"), and spatially autocorrelated random effects ("SE"). Flight configurations are displayed in Fig. 2 of the main manuscript text. Blank entries indicate cases where estimates were numerically unstable or models were otherwise overparameterized.

	4 Flights		8 Flights			12 Flights			
Model	A1	A2	B1	B2	B3	C1	C2	C3	C4
**A. Bearded seals**									
covs	3501	14604	2447	2362	3111	1822	2284	2446	2217
covs + samp	15781	8297	2356	2630	3084	2089	1981	2056	2927
stratum		3249	2509	2436	2606	2073	2020	2104	2311
covs + stratum		33135	2344	2537	2600	2049	2031	2154	2458
stratum + samp		3306	2321	2517	2966	2144	2237	2237	2717
covs + stratum + samp		47513	2346	2461	3380	2105	2194	2134	2536
covs + RE	3734	18108	2471	2334	2873	1920	2046	2137	2287
stratum + RE		3185	2458	2571	2641	2192	2109	2075	2354
stratum + samp + RE		3300	2406	2590	3068	2123	2196	2167	2679
**B. Ringed seals**									
covs	17387	78021	19495	37121	52082	21181	25195	41662	30273
covs + samp	64220	83279	20363	12011	13693	13703	13384	10772	13760
stratum		39055	11164	14496	23951	11421	9904	15102	13777
covs + stratum		45317	11268	13146	16250	9550	8866	12775	11858
stratum + samp		25209	12436	12589	16631	12122	12628	13525	18167
covs + stratum + samp		45152	12959	12563	13216	11119	13730	11682	17943
covs + RE	18754	56089	11791	14862	18727	11972	9581	11638	11291
stratum + RE		43601	10813	14103	24739	11178	8954	14099	15199
stratum + samp + RE		25415	12069	12681	16378	13227	13996	11136	16052
**C. Polar bear**									
covs			341	417	403	285	265	252	309
covs + samp.dens						296	349	265	313
stratum			338	332	448	287	279	267	377
stratum + samp.dens			3090	401	408	292	318	322	309

Table S2. 90% credible interval coverage for the total abundance (N), as estimated from different models (indicated on rows), and as a function of different transect configurations (rows). Each value represents the proportion of $n = 100$ for which the true value of N was between the 5th and 95th quantiles of the posterior predictive distribution for $\hat{N}$. Values close to 0.9 represent "nominal" coverage, while values < 0.9 indicate that estimated variance is likely too low, while values > 0.9 indicate that estimated variance is likely too high. Models could include landscape level covariates (collectively referred to as "covs"), geographical stratum ("stratum"), a measure of sampling intensity (intended as a potential fix for preferential sampling; "samp"), and spatially autocorrelated random effects ("SE"). Different flight configurations are displayed in Fig. 2 of the main manuscript text. Blank entries indicate cases where estimates were numerically unstable or models were otherwise overparameterized.

	4 Flights		8 Flights			12 Flights			
Model	A1	A2	B1	B2	B3	C1	C2	C3	C4
**A. Bearded seals**									
covs	0.95	0.47	0.94	0.98	0.95	0.98	0.95	0.96	0.95
covs + samp	0.83	0.94	0.93	0.89	0.91	0.91	0.95	0.96	0.86
stratum		0.90	0.93	0.89	0.93	0.93	0.94	0.92	0.92
covs + stratum		0.93	0.96	0.88	0.97	0.95	0.94	0.95	0.88
stratum + samp		0.89	0.96	0.90	0.94	0.88	0.90	0.93	0.85
covs + stratum + samp		0.97	0.98	0.94	0.93	0.93	0.94	0.94	0.90
covs + RE	0.95	0.54	0.92	0.97	0.97	0.97	0.92	0.97	0.95
stratum + RE		0.88	0.93	0.89	0.94	0.93	0.94	0.91	0.87
stratum + samp + RE		0.86	0.94	0.93	0.93	0.94	0.94	0.94	0.90
**B. Ringed seals**									
covs	1.00	0.64	1.00	1.00	0.94	1.00	1.00	0.98	1.00
covs + samp	0.93	0.74	1.00	1.00	1.00	1.00	1.00	1.00	1.00
stratum		0.99	1.00	1.00	1.00	1.00	1.00	1.00	1.00
covs + stratum		0.97	1.00	1.00	1.00	1.00	1.00	1.00	1.00
stratum + samp		1.00	1.00	1.00	1.00	1.00	1.00	1.00	1.00
covs + stratum + samp		0.98	1.00	1.00	1.00	1.00	1.00	1.00	1.00
covs + RE	1.00	0.93	1.00	1.00	1.00	1.00	1.00	1.00	1.00
stratum + RE		0.98	1.00	1.00	1.00	1.00	1.00	1.00	1.00
stratum + samp + RE		1.00	1.00	1.00	1.00	1.00	1.00	1.00	1.00
**C. Polar bear**									
covs			0.91	0.89	0.89	0.88	0.90	0.85	0.80
covs + samp.dens						0.93	0.88	0.87	0.85
stratum			0.86	0.85	0.87	0.84	0.87	0.88	0.83
stratum + samp.dens			0.90	0.89	0.88	0.91	0.86	0.92	0.89
